# Ability to control forehand groundstroke of skilled tennis players

**DOI:** 10.1371/journal.pone.0326608

**Published:** 2025-06-24

**Authors:** Ryosuke Furuya, Milos Dimic, Tobias Vogt, Kazuyuki Kanosue, Hiroki Nakata

**Affiliations:** 1 Graduate school of Sport Sciences, Waseda University, Tokorozawa, Saitama, Japan; 2 Institute of Professional Sport Education and Sport Qualifications, German Sport University Cologne, Cologne, Germany; 3 Faculty of Sport Sciences, Waseda University, Tokorozawa, Saitama, Japan; 4 Institute of Health and Sports Science & Medicine, Juntendo University, Inzai, Chiba, Japan; 5 Faculty of Engineering, Nara Women’s University, Nara City, Japan; Ningbo University, CHINA

## Abstract

In tennis, the ability to hit groundstrokes accurately to a target area on the court is important. It is a common belief in tennis coaching that greater concentration leads to more accurate targeting. The purpose of this study was to test this belief by evaluating the variability of the ball-landing position when hitting toward target areas of different sizes and locations. Ten male top-level college tennis players performed forehand strokes and aimed at four target areas: 1) a short area (Short) between the service line and baselines, and 2) a deep area (Deep) just inside the baseline, both with an identical target size (2*2 m); and 3) a large area (Big, 2*4 m), and 4) a small area (Small, 1*1m), both centered in the same location. The average ball-landing position, standard deviation (SD), and bivariate variable error (BVE) were calculated. SD and BVE were normalized relative to the distance between the player and center of the target area. SD in the depth direction (net-baseline direction) was significantly smaller in Short than Deep, but the normalized SDs showed no difference. SD in Big was not significantly different from that in Small, suggesting that it is inherently difficult even for advanced tennis players to intentionally control the distribution of their ball-landing positions.

## Introduction

In tennis, top professional players use forehand strokes more frequently than other shots in matches [1], so the forehand stroke is important in deciding the outcome of a game. To win a game, players must perform accurate forehand strokes, and advanced tennis players can hit more inside the target area than beginners even when they are fatigued [2].

For a simple arm-reaching movement, where subjects extend their arms to point at a target, the accuracy decreases and endpoints exhibit a distribution when visual feedback of the target is unavailable [3,4]. In tennis, such visual feedback is completely useless for controlling each shot, because once players hit a ball, they cannot change its trajectory, even if they notice the trajectory looks different from the one they had planned. Therefore, knowing the distribution of the ball-landing positions is crucial for appropriate tactics and decisions about where to hit the ball in a match, as well as for training in the skill for accurate groundstrokes. However, players and coaches often lack precise knowledge about the exact shape and size of distribution (variability) of ball-landing positions. For example, when aiming at a down-the-line target area with a forehand stroke, the distribution of ball-landing positions can be approximated as an ellipse with the longer axis parallel to the side line [5–7]. According to the results of these studies, controlling shot depth is more difficult than controlling left-to-right variability. However, previous studies analyzed the distribution of groundstrokes only when players aimed at just a single target.

In a real match, players must aim their shots at a specific area in the opponent’s court; the location, distance, and size of the target may vary significantly depending on situations. According to Fitt’s law [8], the speed of a shot directed at a target is influenced by the target’s distance and size. However, there are many situations where players must maintain shot speed, especially in cornered situations (very close to the line). Therefore, players need to quantitatively know the variability of ball distribution in relation to their aiming positions, and understand this variability under given conditions.

A previous study investigated the effect of target distance on backhand shots, and showed that balls aimed at a close target near the service line landed in the target area more accurately than those aimed at a deep target near the baseline; ball speed was also slower when aiming at a closer target [9]. Thus, the closer the distance to the target, the less the variation in ball-landing positions. However, no corresponding studies have examined this phenomenon for forehand shots.

Another problem with previous studies on the distribution of ball-landing positions is the inconsistency in the size of the target area used among studies [5,6,10,11]. When practicing to improve shot accuracy, tennis players typically aim at a single point target rather than a defined area. To the best of our knowledge, no studies have examined the effect of target size on the distribution of ball-landing positions in tennis. Additionally, in tennis coaching, it is a common belief that greater concentration leads to more accurate targeting. Two of the authors in the present study have long experiences as tennis coaches and observed that when a player’s ball-landing positions are widely distributed during practice – especially when aiming at a small target – it is often interpreted that the player lacks concentration on their shots.

The purpose of this study was to clarify how the distribution of ball-landing positions changes in relation to the target area size and distance. Four targets with different sizes and distances were set. First, we set two targets to test the effect of target distance: 1) a short area (Short) between the service line and baseline, and 2) a deep area (Deep) just inside the baseline, both with an identical target size. Additionally, the remaining two targets were set to test the effect of target size: 3) a large area (Big), and 4) a small area (Small), both centered in the same location. Subjects hit the ball, projected from a machine, to each target. We set two hypotheses: 1) as observed for backhand shots [9], the distribution of ball-landing positions would be more concentrated with a closer target (Short) compared with when it is farther away (Deep), and 2) the distribution of ball-landing positions would be less scattered with a smaller target area (Small) compared with when it is larger (Big), because participants may concentrate more in the case of Small.

## Methods

### Participants

Ten male tennis players (age: 19.9 ± 1.2 years; height: 174.7 ± 4.7 cm; weight: 66.1 ± 6.9 kg; experience: 13.5 ± 2.2 years; values expressed as mean ± SD) volunteered to participate in this study. To obtain data on the ability of tennis players with skill levels as high as possible, participants were recruited from the varsity university team, which won the title of All Japan Student Tennis Championship Tournament (intercollegiate competition). Three of them became professional players after graduation. Among them, one player was left-handed, and the others were right-handed. In accordance with the Declaration of Helsinki, the experimental procedure was explained in detail to all participants, and written informed consent was obtained from each one. This study was approved by the Ethics Committee of Waseda University and performed (number 2019−298). The recruitment of participants started on 11.29.2019 and ended on 09.09.2020.

### Procedure

**Fig 1:**
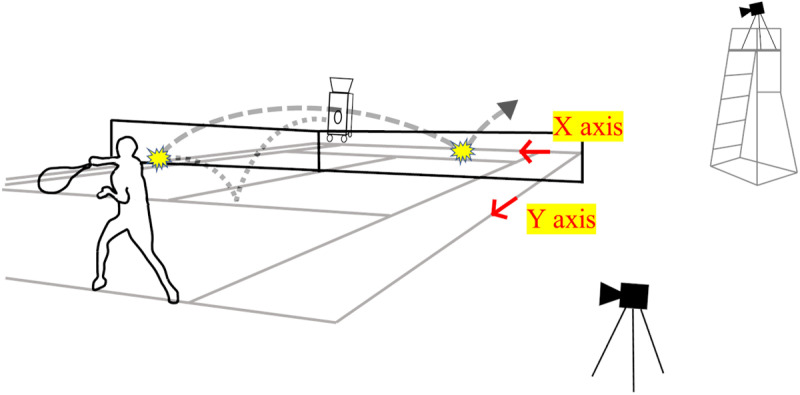
Experimental design for forehand shots. Video images for calculating ball-landing position were captured using a high-speed camera setting on the referee’s table. Balls were projected from a ball machine, and the angle and speed of the returned shots were analyzed using another high-speed camera placed beside the participant.

The experiment was conducted on an outdoor tennis court at Waseda University. Prior to the experiment, participants performed their usual warm-up routines. Each participant was asked to stand on the baseline of the deuce-side and hit forehand shots down the line in a similar manner to the action typically used in matches. The shots were not entirely devoid of spin but involved spin shots with rotation applied to the ball that is generally used by each participant. This tendency was observed across all subjects, and they were told to use the same technique to hit the ball toward each target area. Balls were delivered by a ball machine (Teniser, PM-100, SILVER REED, Japan) set on the deuce side of the opposite court just behind the baseline (Fig 1). The experiment was designed to simulate a real match situation where the players must hit with high accuracy. The speed of the projected ball was about 24 m/s, and balls were delivered at intervals of 4 s.

**Fig 2:**
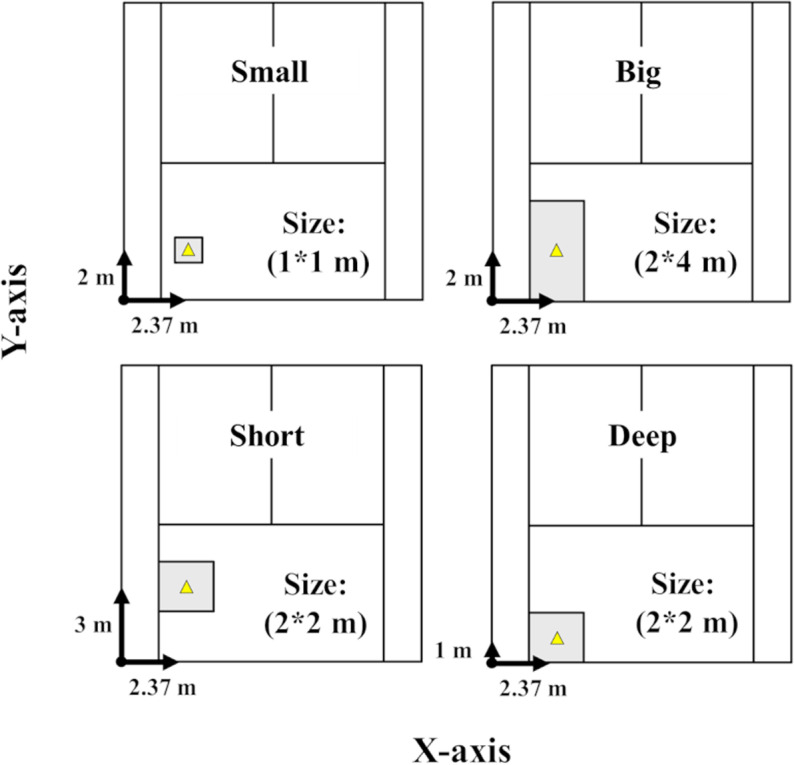
The four target areas: Small, Big, Short, and Deep. Each target area was set up along the singles line on the advantage side. Yellow triangles mark the center of each target area, and arrows indicate the distance from the origin to center.

The participants were instructed to hit the ball into a target area while trying to maintain constant shot speed, regardless of changes in the target [6]. Four target areas with different sizes and locations were set in the opponent’s court (Fig 2). The size of the target was determined with reference to those used in previous studies [5,6,10,11] and finalized at the discretion of one of the authors, who possesses marked coaching experience of tennis. To examine the effect of the target size, big and small target areas were defined. The big area (Big) was a rectangle measuring 4*2 m, located at the corner of the side and service lines. The small area (Small) was a 1*1 m square, centered in the same location as Big. To clarify the influence of the target depth on ball-landing distribution, the short target area (Short; close to the service line of the opponent’s court) and deep target area (Deep; close to the baseline of the opponent’s court) were defined as having the same size (2*2 m).

Participants were asked to hit a total of 200 strokes, aiming at one of the four target areas in the opposite court. Each participant hit 25 balls consecutively at one target area, which was defined as a single set. Two sets were completed for each target area, resulting in a total of 8 sets per participant (2 sets*4 target areas). The order of the target area was randomized for each participant and counterbalanced across all participants. The participants were given a 3-min break between sets to minimize any effects of fatigue. If a participant made a mistake, such as the ball-landing outside the view angle of the video camera (4 m from the center of the target area) or hitting the net, these mistake-trials were excluded from data analysis.

### Measurements

A high-speed video camera (DMC-FZ300, Panasonic, Japan) was located on the side of the advantage court, placed on the referee’s table at a height of 2 m above the ground (Fig 1). This camera was operated at 240 fps and used to record ball-landing positions from the video images.

In order to measure the returned ball speed and angle, another high-speed camera (gc-px1, JVC, Japan) was located to the side of the court with a clear view of the participant (Fig 2). This camera was operated at 300 fps and positioned along an extension of the baseline where the participant was standing. Its field of view covered a 4-meter-wide area centered on the participant.

### Data analyses

**Fig 3:**
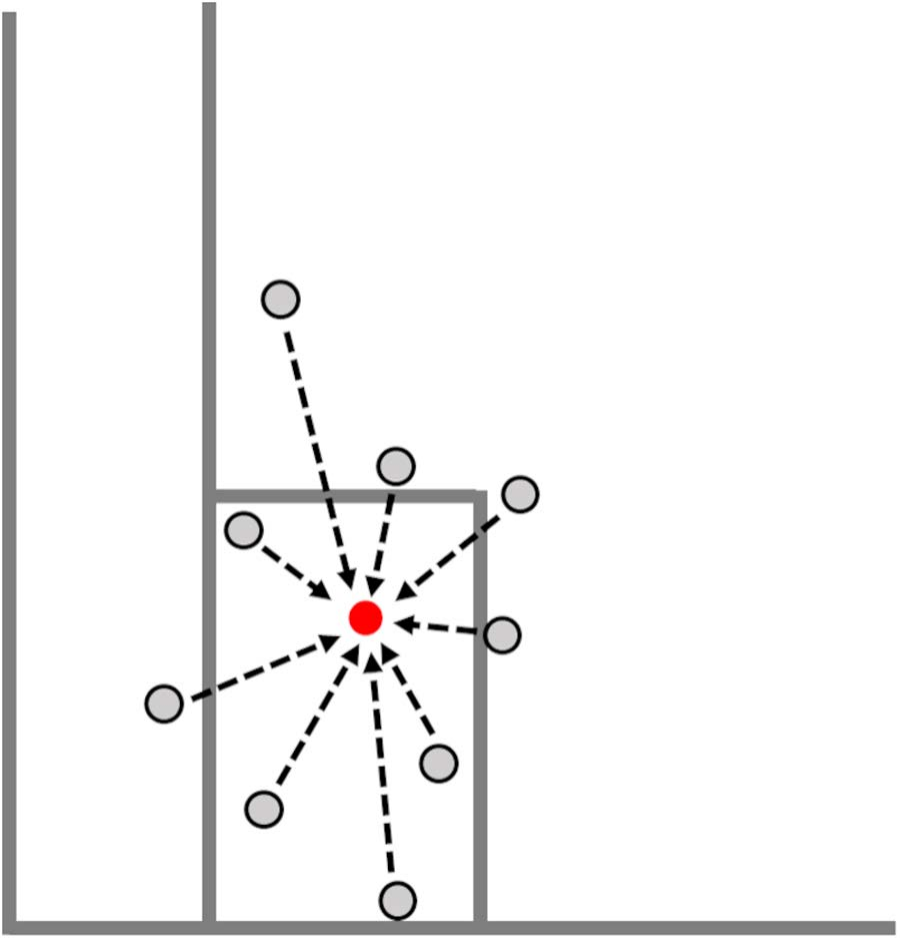
BVE was calculated as the mean position from all ball-landing positions.

To obtain ball-landing positions, the Direct Linear Transformation algorithm [12] was used. Ball-landing positions were manually digitized using software (Frame-DIASⅤ, DKH, Japan). The interaction of the doubles line and baseline on the advantage side was defined as the origin of the coordinates (Fig 2). The X-axis was oriented positively toward the deuce side along the baseline, while the Y-axis was oriented positively toward the net along the doubles line. The average and standard deviation (SD) of the landing positions for both X and Y-axes were calculated for each target area using data from 50 shots. In addition, the normalized SD was obtained by dividing the SD with the distance from the participant’s standing position (baseline on participant’s side of court) to the center of the target area: 21.94 m for Small and Big, 20.94 m for Short, and 22.94 m for Deep. The bivariate variable error (BVE) was also calculated [13] to represent the mean distance from all ball-landing positions (Fig 3).

The normalized BVE was then obtained by dividing BVE with the distance from the participant’s standing position to center of the target. The number of net misses was recorded by the experimenter. Variables were compared between Big and Small, and between Deep and Short, respectively, and not among the four.

**Fig 4:**
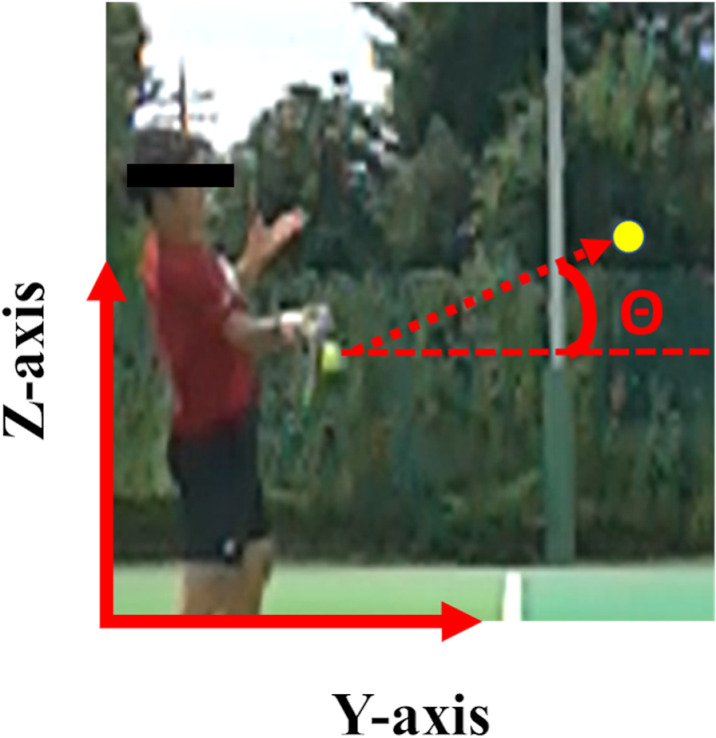
Calculation of ball speed and angle ( **θ).** The returned ball speed and angle were obtained using data at 30 ms after impact. The ball angle (θ) was determined as the angle between the horizontal line and straight line connecting the ball positions at impact with its position 30 ms later.

To obtain the ball speed and angle, the ball was digitized with Frame-DIASⅤ (DKH, Japan) on the lateral vertical plane of the participant’s racket side [14,15] (Fig 4). The origin of coordination was defined as the junction of the singles line and baseline at the participant’s standing position. The Y-axis positive direction was oriented toward the net, while the Z-axis positive direction was oriented vertically upward from the origin. The ball speed was calculated as the average speed over the 30-ms period after the ball’s impact with the racket. The ball angle was determined as the angle formed by a line connecting the ball position at impact to its position 30 ms after impact, relative to the horizontal line (Fig 4). These two variables were averaged across all participants, and separately compared between Big and Small, and between Deep and Short.

### Statistical analyses

Data are presented as the mean ± SD (standard deviation). We analyzed each parameter after checking that data showed a normal distribution using the Shapiro-Wilk test. The accuracy variables (average ball-landing position, SD, BVE, and normalized BVE), average returned ball speed, and average returned ball angle were normally distributed, and compared between two target areas (Small vs. Big, and Short vs. Deep) using the paired t-test. Also, a paired t-test was used to compare SD of ball-landing positions between the X and Y-axis for each target area to clarify the pattern of variation. The number of net misses, which did not follow a normal distribution, were compared using the non-parametric Wilcoxon Signed Rank test. For this comparison, Bonferroni adjustment was applied to the significance level (alpha). Statistical analysis was performed using SPSS software (IBM SPSS Statistic ver. 25.0 for windows; IBM, USA). Significance was set at p < 0.05.

## Results

**Fig 5:**
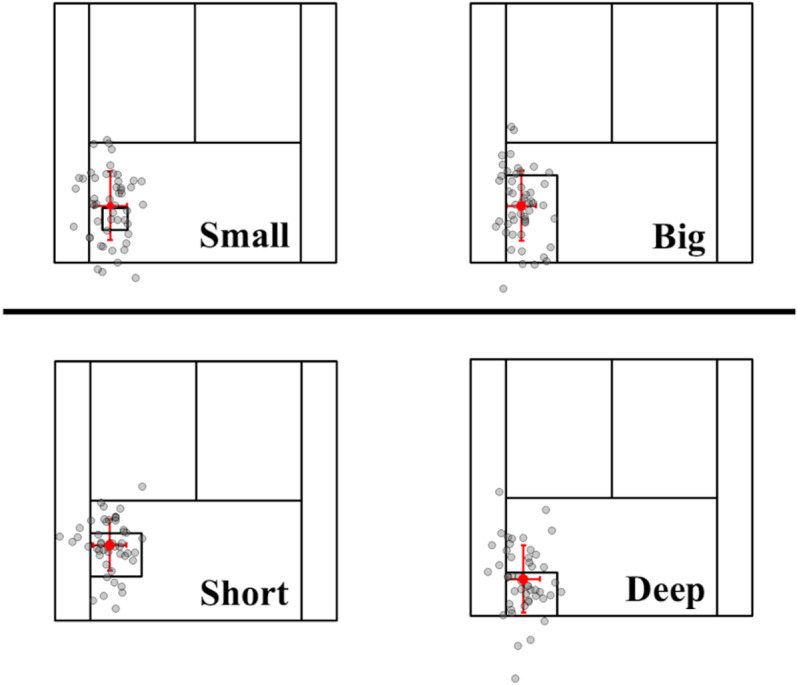
The results from a representative participant show that the distribution of ball landing positions was more scattered in the Y than X-axis.

Fig 5 shows a representative example of ball distribution for the same participant aiming at four different target areas. For all target areas, SD along the Y-axis was significantly larger than that along the X-axis (p < 0.05, respectively).

### Accuracy variables

The number of net misses per 50 trials was 1.4 ± 2.2 for Small, 2.0 ± 2.7 for Big, 2.8 ± 2.9 for Short, and 2.2 ± 2.1 for Deep. There were no significant differences between Short and Deep target areas, or between Big and Small target areas. Also, trials in which the ball landed outside the camera view angle involved only two shots in Deep and Big out of 500 shots across all ten participants.

### Comparison between Small and Big target areas

**Fig 6:**
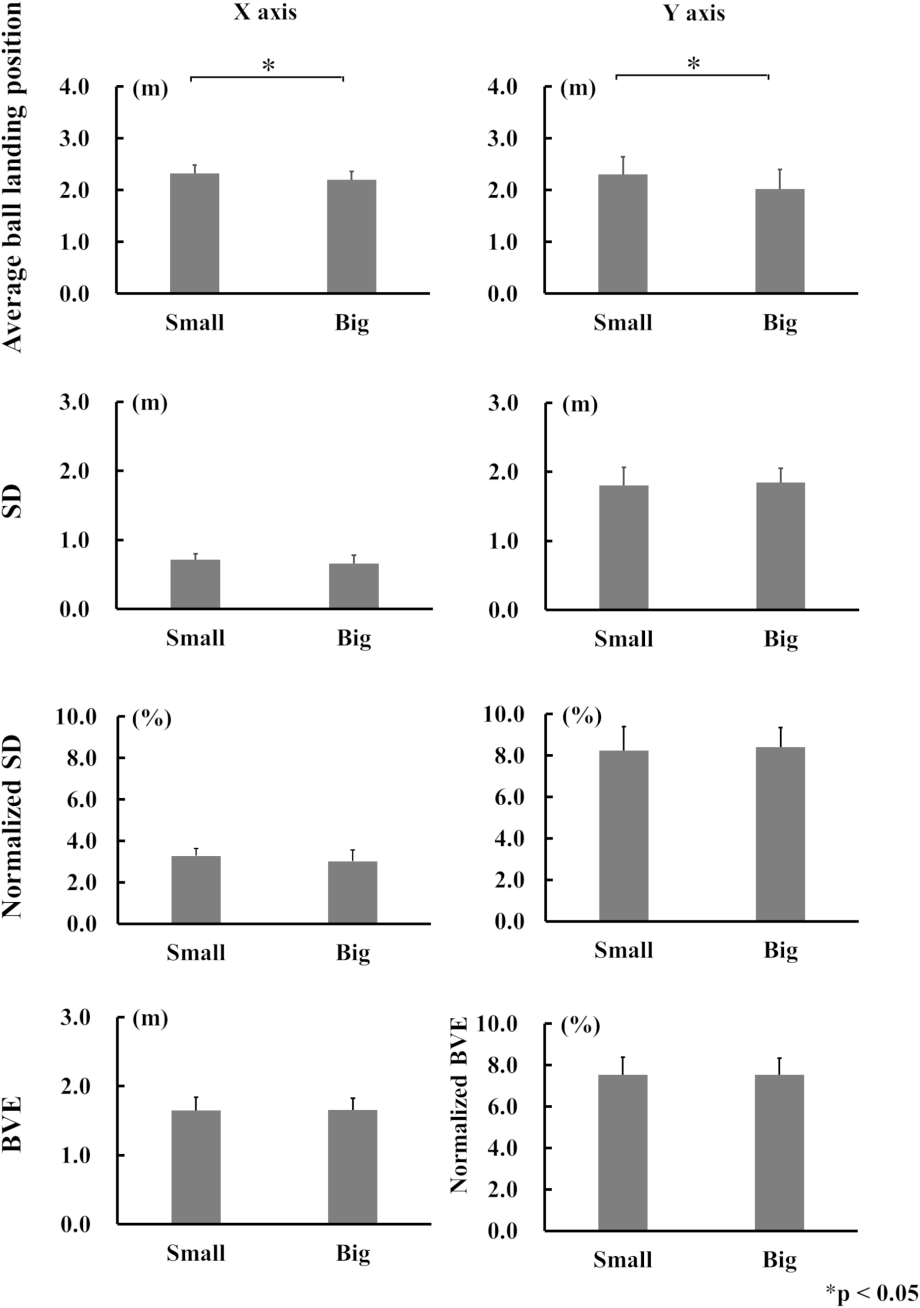
The mean values for each variable in X and Y-axis for Small and Big target areas.

Fig 6 shows the results for each variable for Small and Big target areas. The average ball-landing positions was significantly longer in Small (2.32 m ± 0.16) than in Big (2.20 m ± 0.17) for the X-axis, and in Small (2.30 m ± 0.34) than in Big (2.02 m ± 0.38) for the Y-axis (p < 0.05, respectively). No significant differences were observed between Small and Big target areas regarding SD, normalized SD, and BVE.

### Comparison between Short and Deep target areas

**Fig 7:**
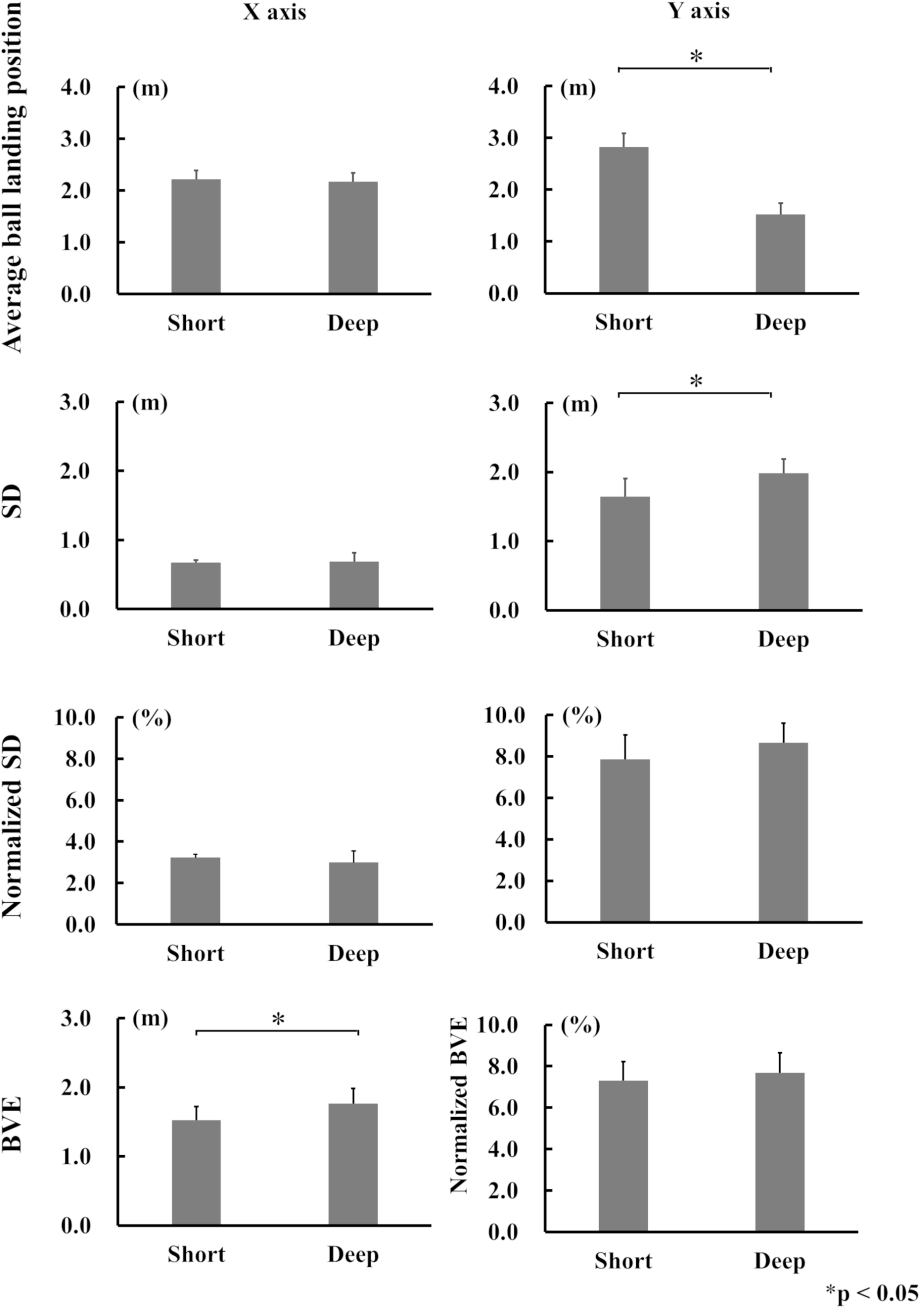
The mean values for each variable in X and Y-axis for Short and Deep target areas.

Fig 7 shows the results for each variable for Short and Deep target areas. The average ball-landing position for the Y-axis was significantly longer in Short (2.83 m ± 0.27) than in Deep (1.53 m ± 0.21) (p < 0.05), while SD for the Y-axis was significantly longer in Deep (1.98 m ± 0.26) than in Short (1.65 m ± 0.24) (p < 0.05). BVE was significantly longer in Deep (1.76 m ± 0.23) than in Short (1.53 m ± 0.19) (p < 0.05). No significant differences were observed between Short and Deep target areas for other variables.

### Returned ball speed and angle

**Fig 8:**
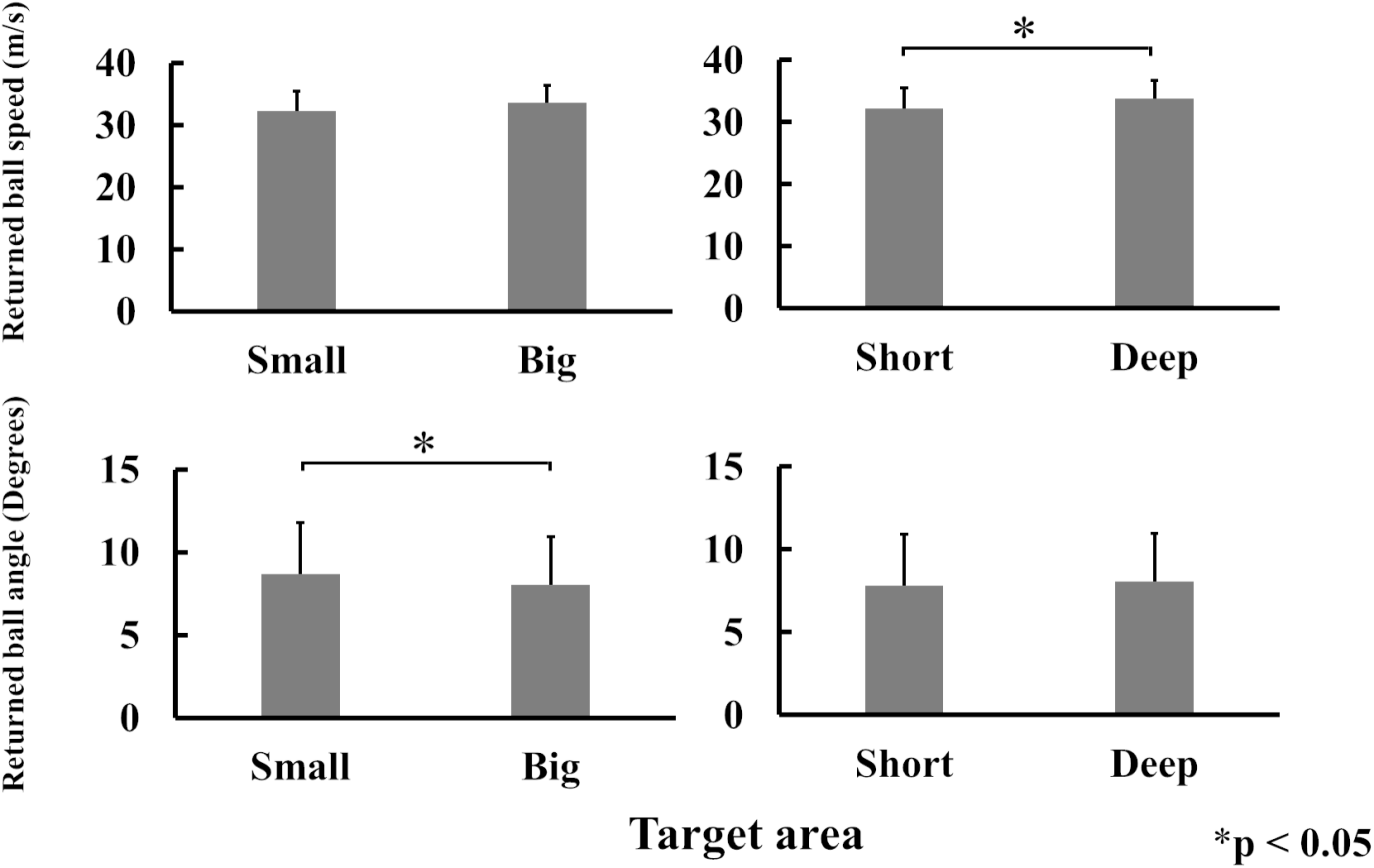
The mean values for returned ball speed and angle for each target area.

The returned ball speed was significantly faster in Deep (33.79 m/s ± 2.88) than in Short (32.17 m/s ± 3.40) (p < 0.05), but no significant differences were noted between Small and Big target areas. The returned ball angle was significantly larger in Small (8.68 degree ± 2.29) than Big (8.03 degree ± 2.05) (p < 0.05), but no significant differences were identified between Short and Deep target areas (Fig 8).

## Discussion

The purpose of this study was to examine changes in the distribution of ball-landing positions when the size and location of the target areas were changed. We firstly hypothesized, that the distribution of ball-landing positions would be more concentrated when the target area was closer to the hitter, and secondly, that the distribution would be smaller when the target area itself was smaller. Our results showed that the distribution of ball-landing positions for Short was smaller compared with the Deep: that is, SD of the Y-axis in Short was significantly smaller compared with that of Deep (Fig 6). Therefore, the first hypothesis was supported. There were, however, no significant differences among SD, normalized SD, nor BVE between Small and Big (Fig 7), meaning that the distribution of ball-landing positions did not differ between Small and Big. Thus, the second hypothesis was not supported.

In the report by Yamamoto et al. (2019), the distribution of ball-landing positions after a forehand shot was elliptical when the player aimed at a target straight ahead, with the long axis aligned parallel to the singles line. This indicates that ball-landing positions varied more in the depth than left-to-right direction. Similarly, in this study, SD along the Y-axis (depth) was larger than that along the X-axis (left-to-right direction), confirming the findings of Yamamoto et al. in all four target areas (Figs 5–7). The greater variation in the depth direction is likely influenced by multiple factors, such as the initial ball speed and angle. In contrast, the smaller variation in the left-to-right direction indicates that it is mainly determined by the initial trajectory [5], leading to less variability in that axis compared with the depth direction. In Small, it is possible that players unintentionally reduced their ball speed slightly in an attempt to aim more accurately at the target or make balls land closer to the net, despite instructions to maintain a consistent ball speed (Fig 8). However, this did not result in reduced variability, because there was no significant difference in variability of the ball-landing position between Small and Big. This suggests that the participants did not consciously adjust the ball angle based on the size of the target area.

The normalized BVE in the present study was around 7.5% (Fig 7). A previous study reported that the distribution of ball-landing positions was less than 5% when normalized by the distance between the server and target [7], using the same indicator as the normalized BVE in the present study. This variation may be attributed to the difference in hitting between ground strokes and services. In ground strokes, it is difficult to always maintain a constant location of impact or ball spin as the ball is hit, even when the ball is supplied by a ball machine. Therefore, hitters must adjust to variables such as the speed and spin of the approaching ball. However, serves are executed under more controlled conditions, allowing players to hit the ball in the manner of their choice. This may explain why the variation in the ball-landing positions was greater in this compared with a previous study.

### Small and Big target areas

A previous study reported that variability in dart throwing does not change based on the target size [16]. The findings of this study were consistent with the previous results, as no significant differences were observed in BVE or SD of landing positions between Small and Big (Fig 6). For Big, a larger variability in ball-landing positions was allowed compared with Small. This suggests that the participants likely focused more on hitting the ball into the target area when aiming at the smaller area. However, the overall distribution of ball-landing positions for Small did not differ significantly from that of Big. This result indicates that the participants aimed at the center of the target area regardless of its size. Even when the target size was reduced to create a situation where larger errors were less acceptable and greater concentration was required, such as attempting a down-the-line shot to the corner of the court in a real match, it was considered challenging, even for advanced players, to consciously reduce the variability in ball-landing positions. The present findings suggest that the distribution of ball-landing positions reflects the players’ genuine ball control abilities rather than their level of concentration. Consequently, the common instruction given by coaches to “Concentrate more to place the ball accurately!” may not effectively improve ball placement accuracy.

Furthermore, tennis players may not have a precise understanding of how accurately they can control a ball. Yamamoto et al. reported that players estimated the distribution of ball-landing positions to be circular, whereas the actual distribution was elliptical [6]. The observation that the distribution of ball-landing positions did not vary regardless of the change in target size does not necessarily mean a lack of effort to aim at the target. The participants of this study were top-level players in Japan. Even for these highly skilled players, the distribution of ball-landing positions could not be intentionally reduced, despite being aware of the target size. This finding underscores the importance, for both players and coaches, of considering the size and shape of ball-landing position distributions for their practices and games.

The returned ball angle was significantly greater in Small than in Big (Fig 8). Nevertheless, there was no difference in the distribution of ball-landing positions between the Small and Big target areas, suggesting that the participants attempted to control their ball-landing positions by increasing ball spin. It is possible that the participants became more focused on accurately placing the ball into the court, and made shots with more spin on the ball, without reducing ball speed. However, since the variation of the ball-landing position did not change, there may not be a significant effect on the ball-landing position even if the ball is hit with more spin.

### Short and Deep target areas

It is noteworthy that there was a significant difference in ball speed between the Short and Deep target areas, accompanied by a change in the Y-axis SD (Fig 6). However, there was no difference in the number of net misses between the two areas, indicating that net misses did not contribute to reducing the Y-axis SD for the Short target area. A previous study demonstrated that national-level tennis players hit backhand strokes more accurately to closer than farther targets [9]. In that study, the ball speed was significantly lower when aiming at closer targets, suggesting that the speed-accuracy trade-off influenced the accuracy of the stroke. The present findings are consistent with the previous observations. When aiming at a shorter target without reducing the ball speed, players may require greater ball spin. It is possible that participants in the present study unintentionally increased ball spin for Short, despite being instructed to “try to aim at the target area but don’t decrease the returned ball speed”. Conversely, for the Deep target area, participants may have tried to increase ball speed to cover a greater distance, resulting in a significant difference in the variability of the ball-landing position distribution between Short and Deep. Additionally, a larger ball angle increases the likelihood of the ball going long, while a smaller ball angle increases the chance of hitting the net [15]. Therefore, when aiming at a closer area, players might aim to increase ball spin without changing the ball angle. However, the present study identified no significant difference in the angle between the Short and Deep target areas (Fig 8), suggesting that the participants adjusted ball spin rather than reduced ball speed. Furthermore, Messier & Kalaska, using a reaching task, reported that endpoint variability reduces as the target distance to the subject decreases, with variations extending along the direction of finger movement [17]. In the present study, the Y-axis distribution of the ball-landing position was smaller in Short than in Deep (Fig 6). This suggests that in tennis, it is simply easier to aim at a closer target than a farther one.

This study did not analyze how variability in the ball’s landing position and distribution change when the returned-ball-speed is intentionally altered. Therefore, further research is warranted.

Since swing mechanics at ball impact would vary among trials even within the same player, it is reasonable to consider the presence of corresponding differences in joint kinematics during preceding stroke execution. Considering that the ball-landing position is the outcome of whole-body coordination and that the scattering of this position would be caused by some fluctuation of body and limb movements, research with a focus on biomechanical and coordination aspects of the human body are of significant importance, which would also be important to obtain information on the risk of lower-limb injury [18].

In this study, balls were fed to the participants by a ball machine to simplify the experimental conditions as much as possible. The distribution of ball-landing positions observed in this study is likely to reflect each participant’s optimal performance. Thus, when returning slow balls, such as those fed manually or stroked with a racket by an expert, a more constrained distribution of ball-landing positions can be expected. Therefore, when returning balls fed manually by an expert, a smaller distribution of ball-landing positions would be expected. However, no data currently support this hypothesis, which represents an important consideration for future research. Moreover, in situations where participants are returning balls hit by a person (opponent player), such as during rallies, it is unlikely that the results of the present study would be contradicted (e.g., it is improbable that the distribution of ball-landing positions for the “Small” target would be smaller than that for the “Big” target). Therefore, under conditions where players are required to maintain shot speed, the conclusion drawn from the present study: players cannot intentionally control the distribution of ball-landing positions, should not be considered specific to machine-fed ball conditions.

## Limitation

Although we tried to recruit as many high-skill-level subjects as possible, we could find only ten. The participants were not world-class players; but not university players. Therefore, there is a possibility that the present findings may only apply to players with a limited range of ability. When a participant was instructed to hit the ball accurately without changing ball speed, the distribution of ball-landing positions remained unchanged. In tennis, especially in a match, players sometimes try to reduce ball speed to control ball placement. Furthermore, it is common to add spin to the ball in order to allow it to clear the net and land promptly on the opponent’s side of the court. In the present study, no specific instructions regarding ball spin were given, nor was spin measured. The distribution of ball-landing positions when players aim at a target without any restriction on ball speed or spin remains to be elucidated and will be investigated in future studies. In this experiment, a ball machine was used to provide participants with balls. It was considered that ball supply by machines would be more stable than that by humans, although no data are available to confirm this. However, with the machine utilized in the present study, balls could be fed to participants so that they had to change their hitting position very little from ball to ball. Although such a situation would not be reproduced in actual tennis matches, it is an interesting topic for future research to assess how the distribution of shots is influenced when players have to change hitting positions for each shot as they do in actual matches.

## Conclusion

This study investigated the distribution of ball-landing positions in top tennis plays when employing the forehand stroke, focusing on two factors: 1) the size of target areas (Big vs. Small) with the same location of the center, and 2) the location of the target areas (Short vs. Deep) with the same size. The results showed no significant difference in the distribution (SD) of ball-landing positions between small and big target areas, even though smaller target areas are generally considered to require greater concentration from players. However, when comparing short and deep target areas, the distribution of ball-landing positions was narrower in the depth direction (net-baseline direction) when aiming at the short compared with deep target. These findings highlight the limitations of accuracy (deviation) even in advanced tennis players. Specifically, even top players cannot intentionally reduce the distribution of the ball-landing positions. Additionally, aiming a ball deeper in the opponent’s court inevitably increases the risk of missed shots. Therefore, during match play, when players have enough time to prepare their strokes, it may be more effective to aim for a slightly shorter area closer to the net rather than targeting near the baseline, in order to minimize the risk of hitting an out-of-court ball. This depends on the shot distribution of each player, and so players should recognize their own abilities to make efficient shots. For example, since clay courts clearly show ball marks, it may be effective for players to assess their own shot distribution by observing the landing positions of several shots.

## Supporting information

S1 Data Individual data. (XLSX)
